# Recurrent Pericarditis, an Unexpected Effect of Adjuvant Interferon Chemotherapy for Malignant Melanoma

**DOI:** 10.1155/2016/1342028

**Published:** 2016-06-22

**Authors:** Farhan Ashraf, Fady Marmoush, Muhammad Ismail Shafi, Ashish Shah

**Affiliations:** ^1^Memorial Hospital of Rhode Island, Alpert Medical School of Brown University, Pawtucket, RI 02860, USA; ^2^Kent Hospital, Warwick, RI 02886, USA

## Abstract

Drug-induced pericarditis is a well-described cardiac pathology that can result from a variety of medications; however, interferon-mediated pericarditis is extremely rare. We present a case of a young female with recurrent pericarditis due to interferon therapy. The role of interferon in adjuvant chemotherapy is well known and yields good effect, but this case highlights the very uncommon phenomena of interferon induced pericarditis and the significant distress it can cause.

## 1. Introduction

Pericarditis is a common disease with many known causes. One such etiology is drug-induced pericarditis, which is a well-described but relatively uncommon cardiac pathology that can result from a variety of medications. Classically implicated drugs include isoniazid, hydralazine, procainamide, dantrolene, doxorubicin, and penicillin. Pericarditis due to interferon therapy, however, is extremely rare. We present a case of recurrent pericarditis due to interferon alpha therapy.

## 2. Case

The patient is a 39-year-old woman with a past medical history of malignant melanoma of the right shoulder (T2N1) with status of postsurgical excision who originally presented to the Emergency Room with a 1-day history of chest pain after receiving her first dose of adjuvant chemotherapy (20 million units/m^2^ intravenous) with interferon alpha. The pain was described as sharp, pleuritic, substernal, and nonradiating. It was exacerbated by lying flat and relieved by leaning forward. Her only associated symptom was shortness of breath. She did not smoke or drink alcohol. Her vital signs were temperature: 97.6 degree Fahrenheit, blood pressure: 104/58 mmHg, heart rate: 80/minute, respiratory rate: 18/minute, and oxygen saturation: 98% on room air. Physical examination revealed regular cardiac rate and rhythm, normal S1 and S2 and no murmurs/rubs/gallops, and no signs of heart failure. Initial evaluation was remarkable for a white count of 2.9 × 10^3^/*μ*L, of which 62% were neutrophils, 23% were lymphocytes, and 14% were monocytes. An electrocardiogram (EKG) showed normal sinus rhythm with an incomplete right bundle branch block without any PR, ST segment, or T-wave abnormalities. No troponin was checked, and she was discharged with a presumptive diagnosis of pericarditis.

**Figure 1:**
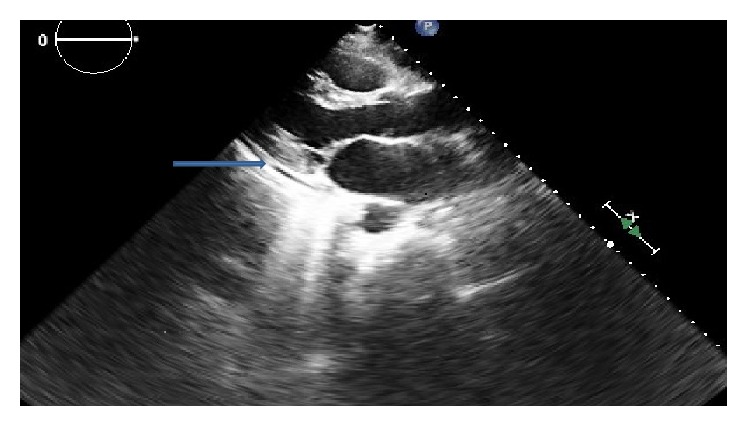
Small pericardial effusion, marked by arrow.

The day after discharge, the patient noted ongoing chest pain, so troponin was checked before her next chemotherapy session and was found to be elevated at 0.323 ng/mL, prompting admission. Her physical exam was unchanged from her initial presentation. EKG demonstrated no significant changes from before. An echocardiogram showed normal systolic and diastolic function with a small pericardial effusion without tamponade physiology (Figure 1). Her troponins downtrended and her telemetry monitoring was unrevealing. She was treated for myopericarditis with colchicine 0.5 mg oral twice a day and ibuprofen 800 mg oral three times a day with which her symptoms improved. She was discharged on two weeks of ibuprofen and three months of colchicine on the above mentioned dose.

Given the suspicion that her myopericarditis was interferon induced, an extensive outpatient discussion involving the patient, her family, her oncologist, and a cardiooncologist took place. Ultimately, given the potential benefits in terms of survival that are conferred by interferon, the decision was made to attempt a rechallenge with the drug.

Two months later she remained chest pain-free, and she resumed the interferon at half the initial dose (10 million units/m^2^). Within ten hours of the reinitiation of interferon therapy, she developed chest pain identical to her previous pain, which was pleuritic and was relieved by leaning forward. Physical examination was again unremarkable with no evidence of a pericardial friction rub or decompensated heart failure. Her EKG demonstrated normal sinus rhythm without PR or ST segment or T-wave abnormalities. Her troponins during this admission were negative. Echocardiogram again showed normal systolic and diastolic function with trace pericardial effusion. She was again treated with ibuprofen 800 oral three times a day and colchicine 0.6 mg oral twice a day, with which her symptoms resolved. She was discharged on ibuprofen for two weeks and colchicine for another three months.

## 3. Discussion

Acute pericarditis is a disease of the fibroelastic sac surrounding the heart. It is the most common disease involving the pericardium. The most common cause for pericarditis is idiopathic, often presumed to be viral or immune-mediated [1]. Drug-induced pericarditis is well-described but is relatively uncommon. Acute pericarditis is diagnosed on the basis of typical pleuritic pain, a pericardial friction rub, suggestive electrocardiogram changes which are typically diffuse ST segment elevations, and a pericardial effusion [1, 2]. The presence of two of the above is considered sufficient to make the diagnosis of pericarditis. Routine testing for viral etiologies is not useful, as the yield for such testing is low, and it usually does not alter management [3].

Interferon is a drug used in many clinical situations, the most common being treatment of hepatitis B and hepatitis C, lymphomas, and melanomas. Its most common side effects include flu-like illness, chest pain, alopecia, rash, pancytopenia, myalgias, and hepatotoxicity. Interferon-mediated pericarditis is extremely rare. Among the different interferons, interferon alpha is known to be the most cardiotoxic. It induces an autoimmune reaction through various mechanisms including production of gamma-globulins and interleukin-6 [4] and inhibition of Allo-specific suppressor T lymphocytes, as well as activation of natural killer cells [5].

Our case demonstrates recurrent, non-dose-dependent acute pericarditis secondary to interferon with a relatively short duration of time to the development of pericarditis. No other typical inciting factors were noted, and withdrawal of the drug resulted in resolution of symptoms. The fact that she developed symptoms so soon after resumption of therapy at reduced dose further strengthens the association between interferon therapy and pericarditis. There are no known predisposing factors that lead to interferon-mediated cardiotoxicity, nor is it thought to be dose-dependent [6]. Based on the limited literature on the subject, the most common cardiovascular side effects of interferon therapy were arrhythmias, ischemic events, and heart failure [7–9]. Previous case reports have documented both pericarditis [10–12] and recurrent pericarditis [13, 14] in the setting of interferon administration, and our case adds to that literature. In cases, pericarditis even leads to development of tamponade fairly quickly [15]. Optimal therapy of interferon-mediated pericarditis is not well-defined, but stopping interferon and treatment with nonsteroidal anti-inflammatory drugs and colchicine results in good recovery.

Our case highlights the importance of suspecting pericarditis in individuals who are receiving interferon therapy when they develop chest pain. It also shows a non-dose-dependent relationship between interferon therapy and pericarditis.

There is not enough information available at this time with regard to interferon induced pericarditis. Based on the limited case repots available, it is difficult to estimate true incidence and ideal treatment of this phenomenon. The mechanism for this pathology is also not defined, making it very hard to predict which individuals are more likely to suffer from pericarditis, apart from those who already had an episode of interferon induced pericarditis.
